# Recent Progress in Phthalocyanine-Polymeric Nanoparticle Delivery Systems for Cancer Photodynamic Therapy

**DOI:** 10.3390/nano11092426

**Published:** 2021-09-17

**Authors:** Wioleta Borzęcka, Adrian Domiński, Marek Kowalczuk

**Affiliations:** Centre of Polymer and Carbon Materials, Polish Academy of Sciences, ul. M. Curie-Skłodowskiej 34, 41-819 Zabrze, Poland; adominski@cmpw-pan.edu.pl

**Keywords:** phthalocyanines, polymeric nanoparticle delivery systems, cancer, photodynamic therapy

## Abstract

This perspective article summarizes the last decade’s developments in the field of phthalocyanine (Pc)-polymeric nanoparticle (NP) delivery systems for cancer photodynamic therapy (PDT), including studies with at least in vitro data. Moreover, special attention will be paid to the various strategies for enhancing the behavior of Pc-polymeric NPs in PDT, underlining the great potential of this class of nanomaterials as advanced Pcs’ nanocarriers for cancer PDT. This review shows that there is still a lot of research to be done, opening the door to new and interesting nanodelivery systems.

## 1. Cancer

Cancer is an uncontrolled growth of cells that can take place in any type of tissue. Unlike normal cells which grow, divide, and die in a conventional way, cancer cells continue to grow and form new, abnormal cells. These cells have the ability to migrate and relocate to other parts of the body from where they started to grow, which is called metastasis. Nowadays, more people than ever before live through early detected cancer.

Cancer was responsible for about 10 million deaths in 2020, making it one of the biggest civilizational health problems, just behind the heart diseases [1]. Among all cancers, breast cancer was the most common in terms of new cases of cancer last year and in general is the most prevalent cancer among women, alone accounting for 30% of female cancers [2]. On the other hand, lung cancer was the most common cause of cancer death, being responsible for 1.80 million deaths in 2020. The next most common were cancers of the colon and rectum (935,000 deaths) and of the liver (830,000 deaths) [1]. Although significant progress has been made to improve cancer treatment over the past decade, the field is still waiting for new breakthrough treatments.

Cancer is a genetic disease caused by changes in genes that control the way cells function, particularly how they grow and divide. The origins of the genetic changes that cause cancer could, e.g., be (1) inherited from parents, (2) increased during a person’s lifetime as a result of errors that occur during cell division, or (3) caused by damage to DNA from environmental exposure (e.g., chemicals in tobacco smoke, ultraviolet rays from the sun) [3].

Tumors are traditionally classified in four ways: (1) by broad classification (e.g., by tissue, organ, and system), (2) by specific type, (3) by grade, according to WHO classifications, and (4) by spread, according to the Tumor Node Metastasis system [3]. In the broad tumor classifications two main categories of cancers can be distinguished: (a) hematologic cancers or blood cancers, which include leukemia, lymphoma, and multiple myeloma (cancers of the blood cells), and (b) solid tumor cancers, which are cancers of any of the other body parts, e.g., breast, prostate, lung, and colorectal cancers. 

Current cancer treatments include chemotherapy, radiotherapy, or surgery, depending on the location and the stage of the tumor. Unfortunately, none of these treatments are perfect. Chemotherapy is a systemic therapy which uses chemotherapeutic agents, special drugs that can damage or destroy cancerous cells but also cause many side effects (e.g., anemia, fatigue, hair loss, organ damage). On the other hand, in radiotherapy malignant cells are controlled or killed by ionizing radiation, which, while it is itself painless, can damage some healthy cells in the area being treated. Finally, in the case of surgery, remaining surgical wounds can be very painful, and swelling or infection may occur at the site of the incision. An alternative to these therapeutic methods may be photodynamic therapy (PDT).

## 2. Photodynamic Therapy

**Scheme 1:**
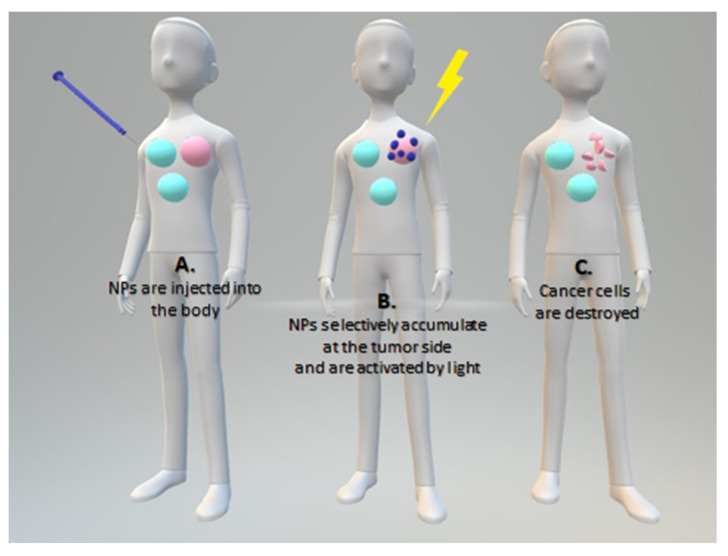
Schematic illustration of the photodynamic action with Pc-NPs in cancer cells (in pink: cancer cells; in aquamarine: health cells; in indigo: Pc-NPs).

Compared to other treatment methods, PDT presents several advantages over conventional therapies because it enables the selective destruction of tumor tissues. This promising therapy combines three components: drugs, light, and oxygen. By themselves these components do not have any toxic effects. When in contact with molecular oxygen and exposed to a particular type of light, the drug agent (sometimes called a photosensitizer or photosensitizing agent or PS), can produce reactive oxygen species (ROS) such as singlet oxygen (^1^O_2_), hydroxyl (^•^HO), peroxyl (^•^ROO) and superoxide anion (^•^O [2]^−^) radicals which lead to the destruction of target cells (Scheme 1). During this process, production of radicals via Type I reaction or more likely the production of ^1^O_2_ (which is the major cytotoxic agent involved in PDT) via Type II reaction can occur [4,5,6,7]. ^1^O_2_ has a short lifetime (10–320 ns) and diffusion range (10 to 55 nm) [8], which make PDT a selective treatment with less secondary effects than other therapies. There are three mechanisms by which PDT can kill cancer cells: (i) the production of ROS kills tumor cells directly; (ii) damage to the tumor-associated vascular system, leading to tumor infarction; or (iii) activation of an immune response against tumor cells. Finally, these three mechanisms can influence each other [4].

Almost 30 years ago, PDT was approved by the Food and Drug Administration (FDA) as a clinical protocol for cancer treatment [4]; however, there are still limitations on its use in all types of cancer, such as low effectiveness in treating large tumors, burns, swelling, pain, and scarring in nearby healthy tissues, and persistent skin photosensitization. It is important to develop new powerful PSs which specifically target cancer cells and can more deeply penetrate tissue to allow treatment of large tumors. In the context of all these limitations, nanoparticles (NPs) have emerged as promising vehicles in PDT, improving cancer treatment [9,10,11,12].

Already many PSs have been applied clinically or preclinically for PDT [13]. Most of the PSs are based on a tetrapyrrole structure, related to the protoporphyrin in hemoglobin [8]. Based on the time of development and specific characteristics of PSs, they are divided into three generations. The first generation PSs are the hematoporphyrins (Hp), to which belongs the first FDA-approved PDT sensitizer, Photofrin^®^ (porfimer sodium) [14]. They are complex mixtures of oligomers, and their intensity of light absorption at the maximum wavelength is quite low. Second generation PSs (porphyrins, chlorins, pheophorbides, bacteriopheophorbides, texaphyrins, and phthalocyanines) have lower skin phototoxicity, higher absorption in the red region of the visible spectrum, and higher purity compared to their first generation precursors. To improve the selectivity, bioavailability, and therapeutic properties of the first and second generation PSs, a third generation was developed. These consist of first and second generation PSs conjugated to biomolecules like proteins, peptides and nanocarriers [15]. Although there are already many first and second generation PSs on the market, the third generation PSs are still under investigation. Hence, development of third generation PSs is an important area of study which could enhance cancer treatment.

There are a few characteristics which a PS should fulfill in order to be considered an ideal PS for PDT; it should: (1) be a photostable and water-soluble chemically pure compound; (2) absorb light in the red or deep red wavelengths to efficiently penetrate tissues; (3) accumulate in the target tissues and rapidly clear from surrounding normal tissues and organs to maximize therapy selectivity; and (4) should not exhibit dark toxicity [8,16,17,18,19]. By “no dark toxicity” it is meant that the only toxic agents responsible for cell death during PDT should be ROS species which are formed when the PS is in contact with molecular oxygen and irradiated with light of the appropriate wavelength. In other words, dark toxicity of PSs occurs during PDT in the absence of illumination, and is an undesirable process [20].

## 3. Phthalocyanines as Therapeutic Agents in PDT

Phthalocyanines (Pcs) are one of the most promising second-generation PSs and meet many requirements for being ideal PSs. Pcs are synthetic porphyrin analogues, consisting of four isoindole units linked together through nitrogen atoms [21]. They have strong absorptions in the visible region, with a maximum of around 700 nm. Unlike porphyrins, Pcs have weak absorption in the range of 400 to 600 nm. This feature is associated with the reduction of skin phototoxicity, which is very often reported as a side effect after PDT treatment with currently approved PSs (e.g., Photofrin and Foscan) [22]. They have large and stable conjugated π-systems, which are good for efficient energy and electron transfer processes. Unfortunately, very often Pcs are hydrophobic species and undergo self-aggregation in aqueous solutions, which drastically reduces their photosensitizing efficiency. 

Pcs, owing to their extended flat hydrophobic aromatic surface, can interact with each other by π-π stacking, acid-base, hydrogen-bond, and donor–acceptor interactions, resulting in self-assembly or aggregation of Pcs in aqueous solutions [23,24]. Two types of aggregations can be distinguished in Pcs: (i) H-aggregation (face-to-face style hypsochromic shift, i.e., blue-shift in absorption), which has a large contact area and strong π-π force; and (ii) J-aggregation (head to tail bathochromic shift, i.e., red-shift in absorption), which has a lower contact area and weaker π-π force [24,25]. In fact, the application of many Pcs in PDT is limited by their aggregation behaviour, which can drastically reduce ROS production.

One of the most successful drugs used for PDT is Photosens, a mixture of sulfonated aluminium phthalocyanines with different degrees of sulfonation (Table 1). It is used in Russia to treat skin, stomach, lip, breast, and oral cancer. Another, silicon-based metallophthalocyanine (Pc 4), underwent Phase I trials for actinic keratosis, Bowen’s disease, and skin cancer [14,26]. The isomeric mixture of di-(potassium sulfonate)-di-phthalimidomethyl ZnPc (Photocyanine) reached Phase II clinical trials in China and demonstrated effective inhibition of human hepatocellular carcinoma HepG2 during PDT treatment [22]. The liposomal formulation of zinc(II) phthalocyanine (ZnPc), CGP55847, was evaluated for the squamous cell carcinomas and successfully completed Phase I/II clinical trials [22]. As stated by Pui-Chi Lo et al. [22], there are many excellent Pcs which exhibit promising potential for PDT but, because of limited financial support from pharmaceutical companies, it is difficult to undergo very expensive Phase II and III clinical trials. Nevertheless, since the perfect PS does not yet exist, there is still a need to improve the photosensitizing efficiency of PSs or create new PSs with better photosensitizing properties. One solution is to combine Pcs with polymer NPs.

## 4. Polymeric Nanoparticle Delivery Systems

NPs used in drug delivery systems include organic NPs (e.g., liposome NPs, polymer NPs, dendrimers), inorganic NPs (e.g., gold NPs, silica NPs, magnetic NPs, carbon nanotubes, quantum dots), and hybrid NPs (e.g., lipid-polymer hybrid NPs, organic-inorganic hybrid NPs, metal-organic framework NPs) [43,44,45].

Among other nanomaterials, polymer NPs have emerged as promising vehicles for PDT (Table 2). The combination of Pcs with polymer NPs can improve their photophysical properties, selectivity for targeted tissues by surface modification, and their low water solubility, and eliminate aggregation of the PSs. Furthermore, such a combination can enhance treatment by increasing blood circulation and selective accumulation in tumor tissues, due to the enhanced permeability and retention (EPR) effect (so-called “passive tumor-targeting”) [46,47]. Compared with normal blood vessels, which have continuous vasculature, tumor vasculature is leaky and can selectively take up NPs with a diameter range of 10 to 100 nm. These particles can stay inside the tumor for a longer time, releasing the drug inside or in the vicinity of the tumor cells [48]. On the other hand, particles which are larger than 100 nm can be cleared from circulation by phagocytes, and particles smaller than 1–2 nm can leak from the normal vasculature [43].

Moreover, the tumor microenvironment is characterized by overexpression of unique cellular markers, i.e., folate or biotin receptors, epidermal growth factor receptors, various integrin receptors, CD44 receptors, glucose transporters, etcetera [92]. Therefore, decoration of nanomedicines with tumor-specific targeting ligands might allow unique recognition and interaction with overexpressed markers resulting in increased accumulation and facilitated internalization of nanomedicines in tumor tissues (“active tumor-targeting”).

**Scheme 2:**
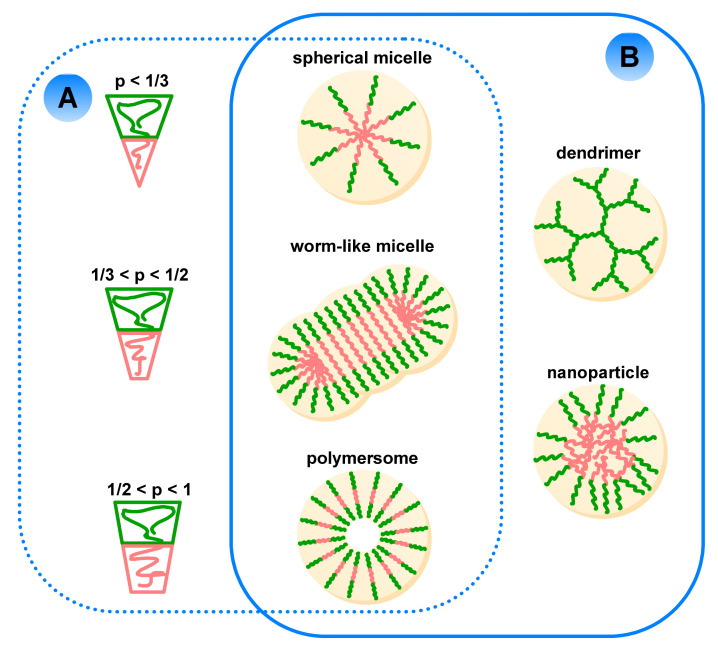
(**A**) Schematic representation of the self-assembled morphologies formed from the self-assembly of amphiphilic block copolymers depending on the packing parameter, p; (**B**) Main polymer-based nanocarriers.

Amphiphilic block copolymers are able to self-assemble in different nanostructures in aqueous solutions. The most common examples are micelles (spherical, cylindrical, worm-like), polymer nanoparticles, and polymersomes (Scheme 2B). By tuning the ratio between the hydrophobic and hydrophilic units of amphiphilic block copolymers and adjusting the reaction conditions in an aqueous solution, distinct polymeric self-assembled structures can be achieved. The parameter which is responsible for the morphology of the self-assembled final product is ‘packing parameter’, p, which can be used to predict resulting self-assembled morphologies (Scheme 2A) [93,94]. It is defined as p = v/a_o_l_c_ (v = the volume of the hydrophobic block, a_o_ = the contact area of the head group, l_c_ = the length of the hydrophobic block) [95]. As a general rule, a packing parameter lower than 1/3 creates spherical micelles, p between 1/2–1 results in polymersomes, and p in the range of 1/3–1/2 gives cylindrical micelles [93,94,95,96].

### 4.1. Polymeric Micelles

Polymeric micelles or aggregates are formed when the self-assembly of amphiphilic block copolymers occurs (Scheme 2B). This reversible process takes place above their critical micelle concentration (CMC) [97]. The hydrophobic part of the block copolymer serves as the micelle’s core, which can encapsulate a hydrophobic drug and protect it against biodegradability. At the same time, the hydrophilic part forms the micelle’s shell, which enables water solubility and prevents aggregation. Depending on (1) the size of the hydrophobic and hydrophilic units of copolymers, (2) the solvent conditions, (3) the water content, and (4) the presence of additives (e.g., ions, surfactants and others), micelles of different morphologies can occur (e.g., spheres, rods, tubules, lamellae and vesicles, which should rather be considered as polymersomes) [98,99,100].

### 4.2. Polymersomes

Polymersomes (also named as polymeric vesicles) and micelles are the most popular and stable morphological structures of amphiphilic polymers in water. Polymersomes are spherical structures made by block copolymers (Scheme 2B). They consist of a polymer-based bilayer membrane with a liquid core that can entrap compounds such as drugs, which are thus isolated and protected from the outside medium. Contrary to micelles, which mostly encapsulate hydrophobic compounds inside their cores, polymersomes can encapsulate hydrophilic molecules within their fluid-filled cores, and additionally entrap hydrophobic molecules within the bilayer walls [101].

As mentioned above, either micelles or polymersomes can be formed depending on the ratio between the hydrophobic and hydrophilic units of copolymers [95]. For instance, Alibilandi et al. [93,94] were able to prepare micelles using the PEG-PLA 5000:5000 copolymer. On the other hand, with PEG-PLA 5000:15,000 the hydrophobic PLA part was too bulky to fit in the corona of a micelle, thus the copolymer formed polymeric vesicles [93,94].

### 4.3. Polymeric Nanoparticles

Polymeric nanoparticles are solid colloidal systems [102] which are formed through physical or chemical processes (Scheme 2B) [103]. Unlike micelles, they do not require an amphiphilic polymer to be prepared [103] and are not characterized by the CMC [102].

Among polymeric nanoparticles, nanospheres or nanocapsules can be distinguished depending upon the formation process [102]. Polymeric nanospheres are matrix-like structures in which the drug can be dissolved, encapsulated, entrapped, chemically bound, or adsorbed on the polymer units. On the other hand, nanocapsules are vesicular-like structures which consist of a single polymeric membrane enclosing a drug-containing liquid core (aqueous or lipophilic) [102,104,105,106,107].

Again, a small difference in preparation method can sometimes result in polymeric micelles or polymeric nanoparticles. As an example, Riley et al. [108] and Heald et al. [109] showed how the physicochemical properties of final polymeric material could be changed just by increasing hydrophobic block length in the starting material. They used a series of poly(lactic acid)-poly(ethylene oxide) (PLA-PEG) diblock copolymers as a model, concluding that as the molecular weight of the PLA block decreased, polymeric micelles were produced. On the contrary, as the molecular weight of the PLA block increased, polymeric nanoparticles were produced.

For the self-assembly of copolymers in aqueous solution into polymeric micelles, polymersomes, or polymeric nanoparticles, the emulsion–solvent evaporation process and the nanoprecipitation process (solvent diffusion) are the most popular. On the other hand, for natural polymers such as albumin, gelatin, or gliadin, the desolvation process is preferred.

The emulsion–solvent evaporation method (single emulsion method, O/W emulsification) is used for drugs which are hydrophobic or poorly soluble in water, and is based on the emulsification process (e.g., ultra-sounds, microfluidizer, high speed homogenizers, etcetera). Two solutions are necessary during method preparation. One is an organic phase: an appropriate amount of the copolymer and the drug or encapsulant dissolved in a volatile organic solvent. This organic phase is then added to a continuously stirring aqueous phase with surfactant, resulting in a stable emulsion. During this process, nano-sized organic solvent droplets are formed to serve as a template for nanocarriers. The organic solvent is then allowed to evaporate at room temperature or under reduced pressure [110]. The double emulsion method (water-in-oil-in-water, W/O/W method) is used to encapsulate hydrophilic drugs into the polymer structure. In a double emulsion, the drug is dissolved in the internal aqueous phase [102]. First, the drug is dissolved in an aqueous phase. Next, the drug solution is added to a vigorously stirring organic phase containing (co)polymer dissolved in a volatile organic solvent. After a water-in-oil primary emulsion is formed, it is added to an aqueous solution under stirring. Finally, the organic solvent is allowed to evaporate [110].

The nanoprecipitation process is a one-step process used to encapsulate hydrophobic drugs in the polymer nanocarrier. Two miscible solvents are required. Ideally, the copolymer and the drug should be soluble in the first (the solvent) and not the second (the non-solvent) [102]. Similarly to the emulsion–solvent evaporation method, two solutions are formed. The organic phase is prepared by dissolving the copolymer and the drug in a polar solvent. Then, the organic solution is added drop by drop to an aqueous phase. The rapid diffusion of the solvent takes place, resulting in the formation of nanovehicles. Unlike the emulsion–solvent evaporation method, no surfactants are needed, and a wider variety of organic solvents can be used (e.g., DMSO, acetone) [102].

### 4.4. Dendrimers

Dendrimers are nano-sized branched synthetic polymers with well-defined, homogeneous, and monodisperse architecture (Scheme 2B) [111]. By reason of their step-by-step controlled synthesis they are related to molecular chemistry. Additionally, because of their repetitive structure made of monomers (dendrons), they are related to polymer chemistry. These molecules were first reported at the turn of the 1970s and during the 1980s [112,113,114]. Since that time, dendrimers have gained a broad range of applications, including in medical fields. They are built of two parts: in the central part, they have as a core a dendrimer, which can be a single atom or a group of atoms. The core is surrounded by dendrons, which are a number of identical fragments. Two approaches to polymeric dendrimer synthesis can be distinguished: (1) the divergent method, pioneered by Tomalia, which follows an exponential-like growth where the synthesis starts from the core of the dendrimer to which the building blocks are attached step by step, and (2) the convergent method, introduced by Hawker and Fréchet, in which dendrons are grown separately and coupled to the core in the final step [111,112,115]. The drug can be encapsulated in the core or conjugated on the surface of dendrimers, making them attractive vehicles for anticancer therapeutics [107,115].

## 5. Phthalocyanine-Polymeric Nanoparticle Delivery Systems for Cancer Photodynamic Therapy

There are already NP-based drug delivery platforms which have been approved by the FDA and others through clinical trials [107]. The first controlled release polymer system for delivery of proteins and other macromolecules was described in 1976 [116]. Since that time polymers have become an important group of materials from which nanomedicines can be developed, and they are already widely used in medical applications, e.g., Gliadel (carmustine-loaded polymer wafers for brain tumor treatment) [117], Zoladex (goserelin acetate dispersed in cylindrical rods of D-L-lactide-glycolide copolymers for hormone-dependent advanced carcinoma of the prostate) [117], Septacin (gentamicin sulfate dispersed into a polyanhydride polymer matrix, for osteomyelitis) [117], Doxil/Caelyx (polymer-coated liposomal doxorubicin for the treatment of AIDS-associated Kaposi’s Sarcoma) [118], and Abraxane (albumin-paclitaxel for breast cancer) [119].

Poly(ethylene glycol) (PEG) is the best known polymer in biomedical applications with “stealth” behaviour. PEG is hydrophilic, non-toxic, and uncharged. Its uncharged properties eliminate the electrostatic interactions with plasma proteins. Its presence in a drug results in prolonged blood circulation times, thus it has been tested in many applications. Already there are natural degradable polymers which are used in PDT, e.g., alginate, chitosan, collagen, albumin, gelatin, and cyclodextrins [120]. Among others, the most popular synthetic polymers used in controlled drug release applications are FDA approved polyesters, i.e., PLA, PGA, PCL or PLGA [121,122].

### 5.1. Pc-PEGylated Delivery Systems in PDT

The clinical success of the first FDA-approved nano-prodrug Doxil^®^ (PEGylated liposomal-doxorubicin) as an anticancer agent has shown how effective a weapon the use of nanomedicine could be in the fight against cancers [123]. Therefore, attaching poly(ethylene glycol) derivatives to the therapeutic agents (PEGylation) is a broadly exploited strategy to confer its “stealth” properties. The PEGylated drugs are characterized by several advantages: (1) prolonged circulation of the prodrug in the bloodstream; (2) increased hydrophilicity; and (3) avoidance of the reticuloendothelial system. Moreover, the PEGylation of phthalocyanine derivatives prevents its self-aggregation, which reduces ROS generation [22]. To date, only a few PEGylated phthalocyanines have been reported as promising PDT agents. 

**Scheme 3:**
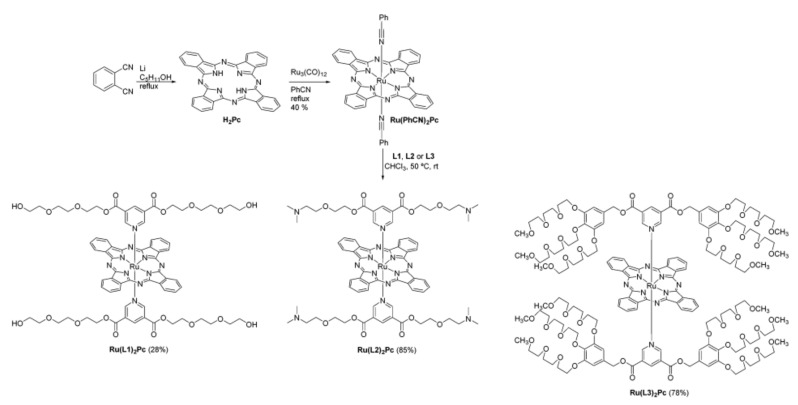
Schematic illustration of the synthetic route of ruthenium(II) phthalocyanines functionalized with 4–12 PEG chains. Reprinted with permission from ref [35]. Copyright 2017 American Chemical Society.

In this field, Torres et al. [35] reported a series of ruthenium(II) phthalocyanines functionalized with 4–12 PEG chains (RuPc-(4–12 PEG) bearing amino, hydroxy, and ether end-groups (Scheme 3) as PDT agents. All synthesized dyes displayed high singlet oxygen generation yields in both DMSO (ΦΔ = 0.76) and water (ΦΔ = 0.48). In vitro cellular studies revealed that the most hydrophilic compound showed the highest phototoxicity despite the lowest cellular uptake. Thus, the authors hypothesized that not only the improved hydrophilicity but also the subcellular localization of photosensitizers played an important role in the activity of photodynamic therapy with these novel photosensitizing agents.

Additionally, Ongarora et al. [36] designed a series of PEGylated ZnPcs with positively charged end-groups, which prevent self-aggregation of free Pcs due to the electrostatic repulsion effect. The non-PEGylated ZnPcs tend to form aggregates visible by atomic force microscopy, while PEGylated ZnPcs dissolve in aqueous solutions with the addition of 1% DMSO. The authors stated that ZnPc with the longest PEG chains exhibited the highest phototoxicity. However, the cellular uptake studies revealed that ZnPcs functionalized with shorter positively charged PEGs had higher accumulation in HEp2 cells compared to ZnPcs with longer PEG chains, although this did not result in better therapeutic outcomes.

Tuncel et al. [29] reported three tetraethylene glycol substituted Zn(II) phthalocyanines (tetra peripheral derivative, tetra non-peripheral derivative, and octa peripheral derivative), and found that the most hydrophilic Pc exhibited the highest ROS generation. In vitro experiments demonstrated that the most hydrophilic ZnPc derivative was the most efficient against colon cancer cells during the phototoxicity experiment. The higher solubility caused less self-aggregation of free Pcs, which resulted in increased ROS generation.

**Scheme 4:**
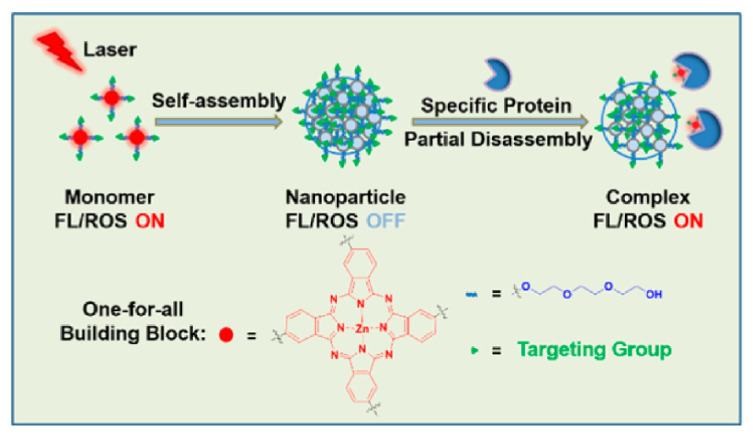
Schematic illustration of the biotin-decorated PEGylated ZnPcs (FL—fluorescence, ROS—reactive oxygen species). Reprinted with permission from ref [124]. Copyright 2017 American Chemical Society.

Furthermore, Li et al. [124] designed and synthesized biotin-decorated PEGylated ZnPcs for lung cancer treatment (Scheme 4). The nanomedicine formed stable nanostructures with an average size of ~100 nm due to the amphiphilic nature of PEGylated ZnPcs. The in vitro cellular uptake studies showed a higher accumulation of nanomedicines in biotin receptor-positive cancer cells (A549, HeLa) compared to biotin receptor-negative cells (WI38-VA13). Moreover, in vivo studies revealed that biotin-functionalized ZnPcs nanostructures had a high level of accumulation in tumor tissues, and after laser irradiation, the inhibition of tumor growth was significantly induced.

**Scheme 5:**
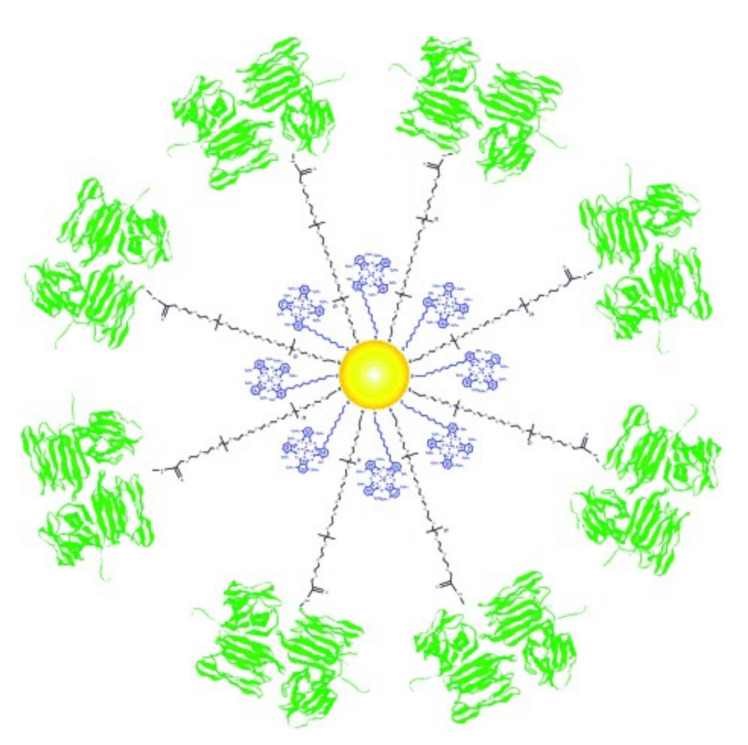
Schematic illustration of the nanomedicine based on PEGylated-Jacalin and PEGylated-dimeric ZnPcs, which were both covalently bonded to gold nanoparticles (T antigen-specific lectin: green; Pc: blue; thiol-functionalized PEG: black; gold NP: yellow). Reprinted with permission from ref [125]. Copyright 2012 Wiley-VCH Verlag GmbH & Co. KGaA, Weinheim.

Lectin peptides make for similar cancer-targeting ligands because of the binding capacity of the Thomsen–Friedenreich (T) carbohydrate antigen, of which overexpression occurs in tumor tissues. Obaid et al. [125] designed and synthesized a nanomedicine based on PEGylated-Jacalin (tetrameric lectin extracted from Jackfruit) and PEGylated-dimeric ZnPcs, which were both covalently bonded to gold nanoparticles (Scheme 5). Extensive in vitro studies demonstrated that jacalin-decorated nanoparticles were able to specifically recognize and interact with T antigen to increase uptake of a photosensitizer. More importantly, after irradiation with 633 nm light, jacalin-decorated nanoparticles possessed significantly higher phototoxicity against HT-29 human colorectal adenocarcinoma cells compared to non-functionalized or lectin-decorated nanoparticles without irradiation.

Likewise, the same group presented NPs based on the same system but functionalized with monoclonal antibodies to target the human epidermal growth factor receptor-2 [126]. The cellular uptake and subcellular localization studies carried out on HT-29 colorectal adenocarcinoma cells and SK-BR-3 breast adenocarcinoma cells revealed no significant differences in both tumor-targeted systems. Moreover, both nanoparticles demonstrated similar levels of ROS production upon irradiation, as well as phototoxicity against cancer cells.

Calavia et al. [127] also reported the preparation of lactose-phthalocyanine functionalized gold nanoparticles for targeted PDT of breast cancer. Lactose is a tumor-targeting moiety taking advantage of breast cancer cells that overexpress galectin-1 receptors. The PEGylated system formed nanoparticles in an aqueous media with an average size of ~3 nm. Compared to the control system (non-functionalized nanoparticles), lactose-decorated nanomedicines were found to selectively target MDA-MB-231 cells overexpressing galectin-1 receptors, and induced a more extensive PDT response. More precisely, the lactose-functionalized nanoparticles induced more cytotoxicity (90%) than the control nanoparticles (61%) post-PDT at the same dose.

Moreover, Darwish et al. [128,129] designed and synthesized a PEGylated-phthalocyanine *star*-polymer photosensitizer possessing a myeloma monoclonal antibody (Daratumumab) and radiolabelled analogues for multiple myeloma treatment. The tumor-targeting PDT nanomedicine was built by using a ZnPc core and four poly(ethylene glycol)_1000_ as branches that were conjugated to ZnPc, followed by conjugation of daratumumab with PEG end groups through facile carbodiimide coupling chemistry (the ZnPc to antibody ratio was 3:1). In vitro cellular studies including quantitative ROS generation, phototoxicity, and cellular uptake demonstrated the potential for applications of this system in myeloma treatment.

The next example of PEGylated phthalocyanines is given by Zhao et al. [37]. They prepared three PEGylated zinc(II) phthalocyanines and showed that these novel Pcs could self-assemble in water to form micelles in the absence and presence of Cremophor EL. Moreover, one of them was encapsulated into silica nanoparticles showing high stability in aqueous media, uniform size, high singlet oxygen generation properties, and high cellular uptake. Photodynamic activity of these systems toward HepG2 human hepatocarcinoma cells was also studied, resulting in enhanced in vitro photocytotoxicity [37].

### 5.2. Pc-Polymeric Nanocarriers Based on Synthetic Polymers in PDT

**Scheme 6:**
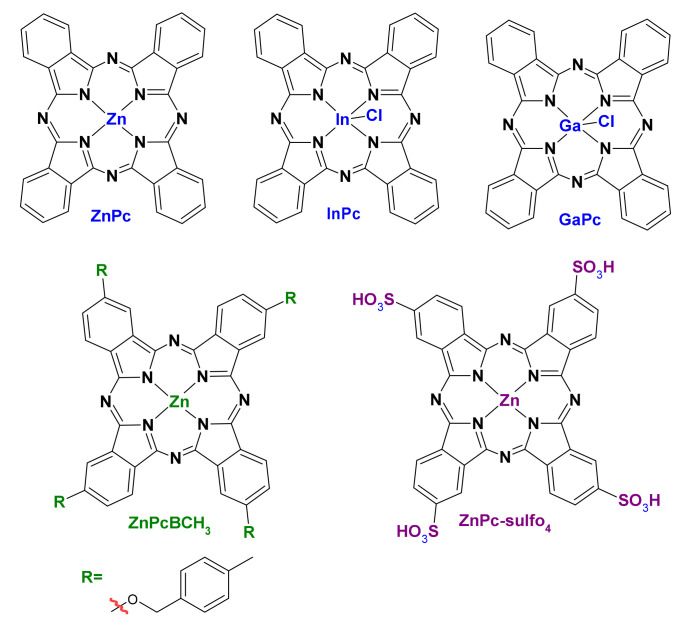
Structures of ZnPc, InPc, and GaPc.

The polymeric nanoparticles derived from biocompatible and biodegradable materials e.g., polylactide, poly(lactide-*co*-glycolide), poly(ε-caprolactone), poly(3-hydroxybutyrate), aliphatic polycarbonates, etc., are broadly studied as drug delivery systems due to their approval by the American Food and Drug Administration (FDA) [130]. Ricci-Junior et al. [49] demonstrated ZnPc-loaded nanoparticles based on bare poly(lactide-*co*-glycolide) with a molar mass of 8000 g/mol (Scheme 6). The nanoparticles had a size of ~285 nm and the in vitro released ZnPc studied showed a slow release of PS, i.e., about 25%, within 10 days. MTT assay has revealed that with a small dose of PS (5 µM), the new nanosystem resulted in 30% cellular viability with no dark toxicity.

Souto et al. [50] reported indium (III) phthalocyanine (InPc, Scheme 6) loaded into nanoparticles consisting of poly(ethylene glycol)-*b*-poly(D, L-lactide-*co*-glycolide) copolymer for breast cancer treatment. Interestingly, the confocal laser scanning microscopy studies showed that free InPc as well as InPc-loaded micelles were similarly internalized into cancer cells. However, aggregation of highly hydrophobic free InPc displayed a reduced ROS level generation. This affected the therapeutic effect because for the same dose of PS, the InPc-loaded NPs reduced the viability of MCF-7 cancer cells twofold compared to free InPc.

Very recently, the same group studied gallium(III)-phthalocyanine (GaPc, Scheme 6)-loaded nanoparticles based on PEGylated PLGA copolymer in order to investigate the PDT efficiency on liver cancer cells and red blood cells [51]. In vitro cellular studies demonstrated that GaPc-loaded nanoparticles display a lower cytotoxicity effect compared to free GaPc. Importantly, nanoparticles loaded with GaPc significantly increased the photodynamic effect on the reduction of the viability of Hepa-1C1C7 cells. In the case of research conducted on red blood cells, the encapsulation of GaPc caused efficient photohaemolysis (83%), compared with free photosensitizer (18%).

Similarly, Mehraban et al. [38] reported the use of PEG-*b*-PLGA-based nanocarriers to encapsulate new phthalocyanine derivatives. A new derivative of phthalocyanine was obtained by incorporating para-methylbenzyoxy groups in the β-positions of the ZnPc periphery (ZnPcBCH_3_, Scheme 6). The modification of photosensitizer does not affect photochemical properties, however it increases solubility in organic solvents, which allowed for efficient encapsulation into nanocarriers. Follow-on cellular studies showed that encapsulated-modified photosensitizer displayed a ~500-fold increase in phototoxicity against cancer cells compared with a free photosensitizer.

De Toledo et al. [52] studied PLGA based nanocarriers coated with an electrolyte layer, in order to increase colloidal stability and facilitate the internalization of nanocarriers for PDT agents. The zinc(II)-phthalocyanine tetrasulfonate-loaded (ZnPcSO_4_, Scheme 6) nanoparticles were coated with a polyalkylamine hydrochloride (PAH) as a weak polycation layer followed by coating with a second layer consisting of poly(4-styrene sulfonate) (PSS) as a strong polyanion, to obtain polyelectrolytic PLGA nanoparticles. MTT tests using B-16 cell lines showed the biocompatibility of nanocarriers and phototoxicity after irradiation, obtaining 90% cell death against 20% for free ZnPcSO4 under the same conditions.

Pound-Lana et al. [34] designed and developed a polyethylene glycol-b-polylactide copolymer with benzyl side-groups to encapsulate chloroaluminum phthalocyanine (AlClPc). The presence of benzyl in the amphiphilic copolymer chain allowed for the attainment of nanoparticles with a high efficiency loading of Pc dyes, owing to the fact that the benzyl side-groups, which were located in the hydroponic nanoparticle core, formed π-π interactions with Pc molecules. However, a major drawback was that physical association by dye π-π interactions resulted in a significant reduction in fluorescence efficiency. The authors concluded that nanocarriers made of PEG-*b*-PLGA are better than those containing side benzyl groups.

**Scheme 7:**
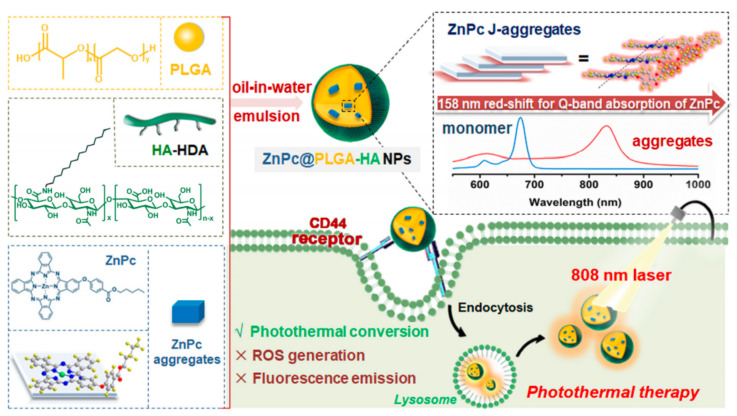
Schematic illustration of ZnPc@PLGA-HA NPs and the action of PTT on CD44-overexpressing cancer cells. Reprinted with permission from ref [53]. Copyright 2020 with permission from Elsevier.

Gao et al. [53] designed and synthesized hybrid nanoparticles consisting of a core of PLGA and an outer shell of n-hexadecylamine-substituted hyaluronic acid (HA) encapsulating a ZnPc-based PS (ZnPc@PLGA-HA NPs) for treatment of solid tumors (Scheme 7). The HA coating was used to take advantage of the overexpression of CD44 receptors to confer the “active-targeting ability” and facilitate the internalization of nanocarriers, whereas encapsulation of ZnPc in these hybrid nanoparticles resulted in encapsulating a zinc(II) phthalocyanine-based shift of the Q-band absorption from 674 nm (free ZnPc in DMSO) up to 832 nm (NP in water). Interestingly, upon light irradiation at 808 nm of the ZnPc@PLGA-HA NPs the photothermal effect was observed instead of the common photodynamic effect. Extended in vitro studies conducted on HT29 and A549 cancer cells (both of which overexpress CD44 receptors) and healthy CD44-negative LO2 cells revealed enhanced photothermal efficacy and selectivity for CD44 receptors. In vivo experiments on HT29 tumor-bearing nude mice showed tumor ablation upon laser irradiation, demonstrating high potential for clinical application.

Another widely studied polyester in terms of the encapsulation of biologically active substances is polylactide, because of its excellent biocompatibility. Wilk et al. [54] have reported an interesting example of employing a poly(ethylene glycol)-*b*-poly(L-lactide) copolymer (mPEG-*b*-PLLA) for encapsulation of ZnPc. The confocal laser scanning microscopy (CLSM) studies revealed an enhanced cellular internalization of ZnPc-loaded micelles in metastatic melanoma cells (Me45) compare to control normal keratinocytes (HaCaT). The comprehensive cellular studies showed excellent biocompatibility, that is, lack of cytotoxicity against macrophage, endothelial cells, and low hemolytic activity. Moreover, the apoptotic assay demonstrated effectiveness in inhibiting cancer cell growth, with a better reaction against metastatic melanoma cells. 

Interestingly, the same group studied the localization of encapsulated three Pcs (ZnPc and its tetrasulfonic acid (ZnPc-sulfo_4_) and perfluorinated (ZnPcF_16_) derivatives) of varying hydrophobicity in PEG-*b*-PLLA micelles [131]. ZnPc was localized within the hydrophobic micelle core, whereas both ZnPcF_16_ and ZnPc-sulfo_4_ were in the hydrophilic micelle PEG corona. They concluded that localization of PS in nanocarriers plays an important role with respect to the photochemical properties, whereas the biggest difference compared to control free phthalocyanine was observed when it was in the core of the micelle. For the Pc localized in the micelle core, the photostability and ability to generate ROS was most significant in the micellar solution compared to free Pc. Thus, the cargo locus is an important feature for the photochemical properties of the PSs.

As a subsequent step, the same group reported tumor-targeting folate-decorated PEG-*b*-PLLA micelles loaded with ZnPc, which demonstrated enhanced drug uptake within cancer cells and thus resulted in better therapeutic outcomes [55].

Conjugation of PS to an amphiphilic copolymer is one approach to improving the stability and overall photochemical properties of PS. Wilk et al. [56] developed a facile method to synthesize the ZnPc conjugates with PEGylated Pluronic P123 and PLLA copolymers whereby amphiphilic constructs self-assemble into biodegradable micelles with superior stability and biocompatibility. Preliminary biological assays indicate that both ZnPc-functionalized micelles are internalized into tumor cells, and possess better photodynamic activity compared to free ZnPc.

In a similar context, Conte et al. [58] studied the potential of a system using nanoparticles as PS carriers through the skin. For this purpose, the ZnPc was encapsulated in PEG-*b*-PCL micelles assisted with 2-hydroxypropyl-β-cyclodextrin (HPβCD). The HPβCD was not chemically or physically linked with nanoparticles but rather were added to the nanocarrier mixture to increase the skin penetration of ZnPc-loaded nanoparticles. The transport studies of PS through porcine ear skin revealed that free ZnPc-loaded NPs allowed to delivery of PS to the stratum corneum, whereas ZnPc-loaded NPs assisted with HPβCD were capable to penetrate into much deeper layers of the skin. Fluorescence imaging experiments showed that HPβCD caused an alteration of the water profile in the skin, causing a reduction of the degree of hydration at the stratum corneum/viable epidermis interface, which significantly increases the nanocarriers’ permeation.

**Scheme 8:**
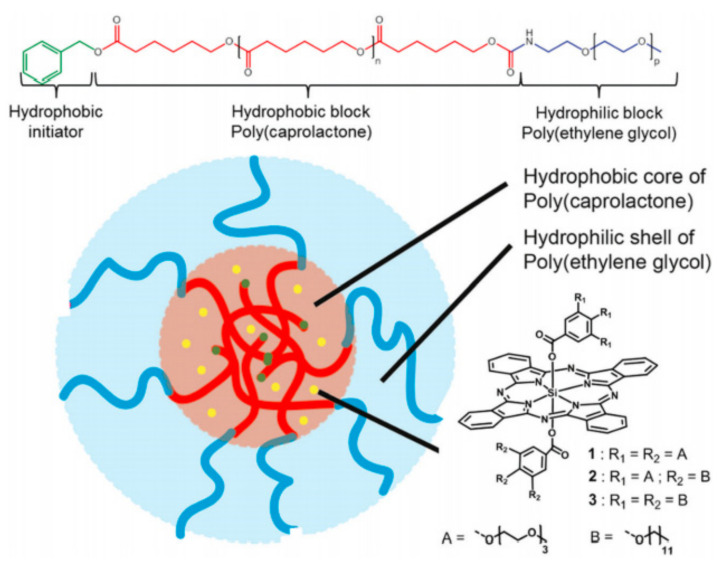
Schematic representation of the PEG-*b*-PCL based micelles as carriers for a series of new third generation PS based on SiPc (compounds **1** and **2**, while **3** serves as a reference lipophilic derivative) in PCL–PEG polymeric micelles. Reprinted with permission from ref [40]. Copyright 2020 The Royal Society of Chemistry.

The PEG-*b*-PCL based micelles are highly promising nanocarriers for drug delivery due to their excellent biocompatibility and biodegradability. Therefore, they have also been used as nanocarriers of PS. Very recently, Torres et al. [40] reported PEG-*b*-PCL based micelles as carriers for a series of new third generation PS based on silicon phthalocyanine (SiPc) (Scheme 8). The two target SiPc compounds bear two axial benzoyl substituents, each of them with either three methoxy(triethylenoxy) chains, or one with three methoxy(triethylenoxy) chains and one with three dodecyloxy chains (Scheme 8). 

Both novel SiPc derivates are amphiphilic, with a hydrophobic core, while lipophilicity increases along with modifications allowing for correlation of the loading properties in the micelles due to hydrophobic/hydrophilic balance of PS structure. The authors claimed that TEM and DLS characterization of these new micelles was impossible because of their small size and the absorption of the DLS wavelength by the PS during DLS measurement. Although lacking full characterization, in vitro cellular experiments with these new nanomaterials showed the great efficacy of this third generation nano-photosensitizer system in PDT. However, in vivo experiments are still necessary to confirm its clinical potential.

Xiao et al. [59] described the synthesis of prop-2-ynyloxybenzyloxy axially or peripherally substituted zinc and silicon phthalocyanine derivates, which were subsequently encapsulated into mPEG-*b*-PCL micelles. Interestingly, the modification in the axial positions reduced aggregation of PS in aqueous media better than that at the peripheral position. Both types of PSs were effectively encapsulated into micelles and were internalized into cancer cells. However, the SiPc derivates displayed higher intracellular ROS generation compared with ZnPc derivates, resulting in better photocytotoxicity against MCF-7 cells.

Asem et al. [60] conducted research using PEG-*b*-PCL based micelles loaded with aluminum phthalocyanine (AlPc). A series of PEG-*b*-PCL copolymers were used as a nanocarrier matrix to confer the high PS loading efficacy and controlled release performance for optimal PDT efficacy. Extensive in vivo and ex vivo studies using fluorescence imaging have shown increased biodistribution and organ uptake of the encapsulated PS compared to free PS, clearly emphasizing the advantages of AlPc-loaded nanoparticles.

Pluronics are a class of biocompatible commercially available amphiphilic copolymers approved by the FDA for biomedical applications. It has a triblock PEO–PPO–PEO structure (PEO: poly (ethylene oxide) and PPO: poly(propylene oxide)). Due to their amphiphilic nature pluronics form stable NPs with a hydrophobic PPO core and a hydrophilic PEO outer corona. In order to develop a biocompatible efficient PDT colloidal system, Py-Daniel et al. [61] reported pluronic F127-based micelles incorporating AlPc. Pluronic F127 loaded with PS formed highly stable micelles with high loading efficiency, i.e., 90%. Preliminary cellular studies conducted on A549 human lung carcinoma cells demonstrated that light irradiation (660 nm LED, fluence of 25.3 J/cm^2^) for 18 min resulted in cellular damage up to 90% for low dosages of PS (0.1–5.0 lgmL^−1^). Additionally, the lack of overall system cytotoxicity and the fact that pluronics are commercially available combine to make this platform suitable for clinical applications. 

Recently, Motloung et al. [62] reported a series of novel benzothiazole and tetra-pyridyloxy functionalized indium (III) and zinc (II) phthalocyanine loaded into Pluronic F127 and Pluronic L121/F127 mixed micelles. They studied the photophysicochemical behavior and PDT activity for free PSs and those encapsulated in micelles. Indium (III) phthalocyanines displayed better PDT activity compared to the corresponding ZnPc analogues. Moreover, the PDT activity of studied PSs was significantly higher when PSs were loaded in micelles. Chiarante et al. [63] found a similar effect with ZnPc derivative-loaded polymeric poloxamine micelles consisting of commercially available copolymer Tetronic^®^ 1107 for colon carcinoma treatment.

**Scheme 9:**
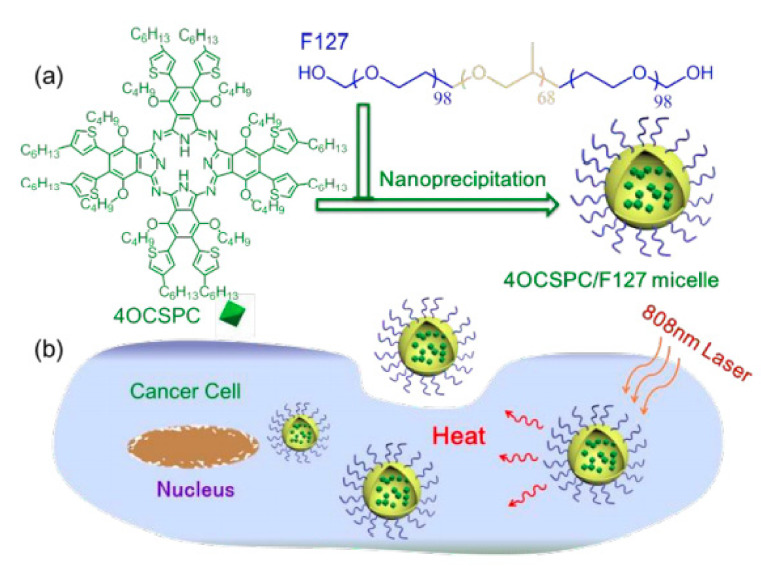
Schematic illustration of the 4OCSPC-based micelle for PTT. (**a**) The structure of 4OCSPC and preparation of 4OCSPC/F127 micelle. (**b**). PTT action the new micelles. Reprinted with permission from ref [64]. Copyright 2019 The Royal Society of Chemistry.

Furthermore, Li and associates [64] reported pluronic F127-based micelles for encapsulation of a novel lipophilic Pc molecule (4OCSPC) for photothermal cancer therapy (PTT, Scheme 9). This system possessed strong absorption in the deep NIR region (808 nm), resulting in a superior photothermal activity. In vivo studies conducted on mice bearing 4T1 tumors showed that micelles accumulated in tumor tissues and, when subjected to 808 nm laser irradiation, improved the growth inhibition effect. More precisely, after the internalization of micelles in tumor tissues during exposure to 808 nm NIR laser for 10 min, the temperature of the solid tumor site increased from 37.8 °C to 59.4 °C, resulting in a tumor growth inhibition rate of 90%. Very recently, the same group has improved the 4OCSPC/F127 nanosystem to achieve a dual PTT and chemotherapeutic effect [65] The 4OCSPC/F127 micelles were incorporated into thermo-sensitive poly(N-isopropylacrylamide) (pNIPAM)-based hydrogels. The final composite hydrogel was also loaded with doxorubicin, with remote-controlled drug release triggered upon 808 nm laser irradiation. In vitro studies revealed that joint action of photothermal performance of 4OCSPC and doxorubicin exhibited a synergistic effect for inhibiting the proliferation of HeLa cells, vide infra.

Other promising methods to improve the stability and effectiveness of PDT agents are nanocarriers based on vinyl copolymers obtained via controlled radical polymerization (e.g., ATRP, RAFT SET-LRP). In this context, Feuser et al. [66] reported ZnPc loaded poly(methyl methacrylate) (PMMA) NPs as PDT agents for leukemia treatment. Obata et al. reported a series of polystyrene-*b*-poly(polyethylene glycol monomethyl ether acrylate) (PSt-*b*-PPEGA) [67] and poly(N-substituted acrylamide)-*b*-poly(polyethylene glycol monomethyl ether acrylate) (P(R)-*b*-PPEGA) [68] copolymers synthesized via RAFT as nanocarriers for ZnPc for PDT. Both systems displayed the light-dose-dependent cytotoxicity of ZnPc-loaded micelles in cancer cells, suggesting a promising photodynamic effect for hydrophobic PS loaded in micelles. B. Vilsinski et al. [69] demonstrated micelles based on poly(styrene)-*b*-poly(acrylic acid) (PS-*b*-PAA) as a chloroaluminum phthalocyanine delivery vehicles. In this area, the team of Zhu et al. [132,133] synthesized a series of homo-, block-, or random copolymers functionalized with ZnPc moiety in side-chains via RAFT. Among them, PS-modified copolymers self-organized into nanoparticles that possessed significantly increased singlet oxygen quantum yields compared to free PS. 

In another example, Yu et al. [70] developed a poly(ethylene glycol)-poly [2-(methylacryloyl)ethylnicotinate] (PEG-PMAN) polymer with aromatic nicotinate nanoparticles to load ZnPc as a PDT agent for the PDT of osteosarcoma. The authors claimed that the interaction of aromatic nicotinate with ZnPc contributed to the efficient loading of ZnPc into micelles. In vitro experiments revealed that ZnPc-loaded PEG-PMAN micelles were well internalized and were able to significantly increase ROS production in osteosarcoma cells after red light irradiation. This resulted in a mitochondrial injury which followed the apoptosis of cancer cells. The cytotoxicity of micelles was 100-fold better compared to free ZnPc. Moreover, in vivo study showed strong inhibition of tumor growth 14 days after PDT treatment, suggesting the promising clinical potential of this system in PDT for osteosarcoma.

In addition, Gupta et al. [71,72,73] loaded Pc 4 into biocompatible poly(ethylene glycol)-*b*-poly(ε-caprolactone) block copolymer micelles which were surface-modified with epidermal growth factor receptor (EGFR)-targeting GE11 peptides. Thus, the final material could actively target EGFR-overexpressing cancer cells in vitro. They were able to prove that in comparison with non-targeted formulations, the EGFR-targeted Pc 4 nanomaterial possessed higher intracellular uptake. Additionally, it showed a much better PDT response after photoirradiation, within shorter periods of time compared to non-targeted formulations.

Simioni et al. [42] encapsulated photosensitizer silicon tribenzoporphyrazinato with two n-dimethylaminoethanoyl as axial groups on silicon (NzPc) into PLGA nanoparticles. This new material increased the singlet oxygen production compared to free PS. The in vitro experiments also showed its superiority over free PS.

Peng et al. [134] observed the aqueous aggregation tendency in 1–2 generation poly(benzyl aryl ether) dendrimer zinc Pcs with cyano- and carboxylic-terminal groups. Since it is known that one of the solutions to avoid aggregation of Pcs is to attach dendritic units to the axial position of a ruthenium or silicon centre, they decided to prepare 1–2 generation poly(benzyl aryl ether) dendrimer silicon Pcs with axially disubstituted cyano-terminal functionalities [74]. To increase solubility and accumulation selectivity at tumor sites they encapsulated novel dendrimer silicon Pcs into three amphiphilic PEG–PCL diblock copolymers with different hydrophilic/hydrophobic proportions. Finally, they studied the effect of dendritic generation and hydrophilic/hydrophobic proportion of PEG-*b*-PCL diblock copolymers on the photophysical properties of dendrimer Pcs. Interestingly, the first-generation dendrimer silicon Pc had higher fluorescence and longer lifetimes than the second-generation dendrimer silicon Pc. Additionally, PEG_2000_–*b*-PCL_4000_ copolymer was recognized as a promising nanocarrier for delivery of PS.

The same group [75] encapsulated tetra(4-sulfoazophenyl-4′-aminosulfonyl) chloride aluminum phthalocyanine (S-AlPc) into micelles of amphiphilic triblock copolymer, PLL-*b*-PEG-*b*-PLL (poly(L-lysine)-*b*-poly(ethylene glycol)-*b*-poly(L-lysine)) and micelles of diblock copolymer, MPEG-*b*-PLL methoxypoly(ethylene glycol)-*b*-poly(L-lysine). The cellular uptake and photoactivity of S-AlPc were greatly improved by encapsulation into PLL-*b*-PEG-*b*-PLL.

More recently, Ma et. al. [41] synthetized benzyl ester dendrimer silicon phthalocyanine (D-SiPc) and encapsulated it into amphiphilic block copolymers, methoxypolyethylene glycol-b-polylactic acid, MPEG_5000_-b-PLA_3000_, to form polymeric micelles. These exhibited high photo-cytotoxicity toward U251 glioma cells under laser irradiation, proving their potential for PDT.

Huang et al. [39] prepared a series of nanocarriers formed via electrostatic interaction between the periphery of negatively charged 1–2 generation aryl benzyl ether dendrimer zinc (II) phthalocyanines and positively charged poly(L-lysin) segment of the triblock copolymer, poly(L-lysin)-*b*-poly(ethylene glycol)-*b*-poly(L-lysin) (PLL-*b*-PEG-*b*-PLL). Interestingly, the singlet oxygen quantum yields of free Pc and its nanoformulations exhibited generation dependence. Moreover, the intracellular uptake of dendrimer Pcs encapsulated into micelles in HeLa cells was better than uptake of free PS. The photocytotoxicity of Pcs incorporated into polymeric micelles was also increased.

**Scheme 10:**
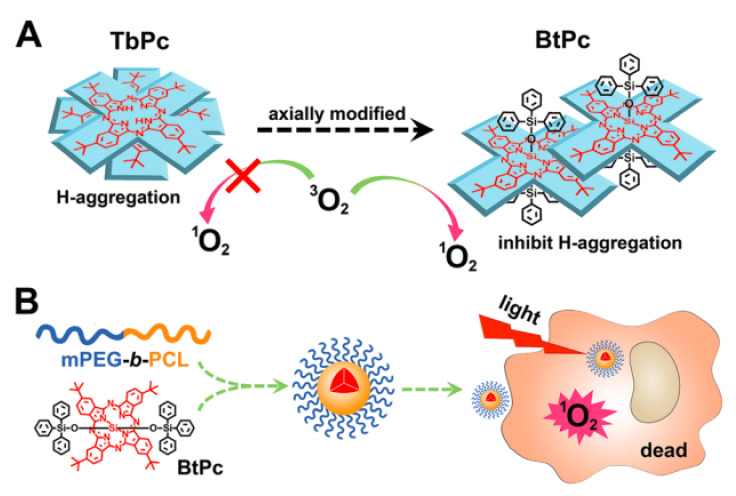
Schematic illustration of: (**A**) the aggregation patterns of different phthalocyanines and their effect on ^1^O_2_ generation; (**B**) self-assembly of mPEG-b-PCL and BtPc for PDT. Reprinted with permission from ref [25]. Copyright 2018 The Royal Society of Chemistry.

Additionally, the group of Guo and Shi et al. [25] axially modified TbPc (tetra(4-tert-butyl)-phthalocyanine), resulting in an axial modified phthalocyanine BtPc (bis-triphenylsilyloxy-silicon-tetra(4-tert-butyl)-phthalocyanine) (Scheme 10). Thanks to this technique they avoided unwanted H-aggregation, which hampers ROS production. Because of their water non-solubility, BtPc and TbPc were encapsulated into methoxy-poly(ethylene glycol)-b-poly(ε-caprolactone) polymeric micelles. BtPc showed a strong singlet oxygen generation ability, and in vitro studies proved that the BtPc loaded polymeric micelles possessed better photodynamic therapy efficiency on HeLa cells than corresponding micelles with non-axial modified phthalocyanines.

### 5.3. Polymeric Nanocarriers Based on Natural Polymers

Biodegradable natural polymers are investigated extensively as nanovehicles to encapsulate and deliver a variety of drugs in cancer treatments. There are many types of natural polymers, e.g., polysaccharides, proteins, peptides, collagen, albumin, gelatin, alginate, and fibroin [135]. 

Chitosan is a polysaccharide with 2-deoxy-2-(acetylamino) glucose units bonded by 1,4-glycosidic linkages. Recently, chitosan and its derivatives have been studied as nanovehicles in the pharmaceutical field due to their biocompatibility and low toxicity, and the ability of amphiphilic chitosan to increase the water solubility of hydrophobic drugs. Moreover, chitosan can self-assemble into core–shell nanoparticles [80].

To illustrate this, Yang et al. [80] prepared ZnPc-loaded positively charged amphiphilic phosphonium chitosan nanomicelles, observing an enhancement of PDT efficacy. First, N-acetyl-L-phenylalanine-(4-carboxybutyl) triphenylphosphonium bromide chitosan (CTPB-CS-NAP) was synthesized. Then, ZnPc was encapsulated in CTPB-CS-NAP. The introduction of cationic groups to chitosan molecules effectively improved its water solubility. The in vitro cellular uptake and PDT experiments revealed the excellent phototoxicity of ZnPc chitosan nanomicelles, with no dark toxicity observed.

Likewise, De Souza et al. [79] prepared three ZnPc loaded polymeric nanocapsules from chitosan, PCL and PCL coated with chitosan and studied their photodynamic activity, photostability, and drug release. Although the new materials presented promising properties for PDT, the in vitro and in vivo behaviour have yet to be determined. In more recent work, the same group [136] observed enhancement of PDT efficacy of chloroaluminum phthalocyanine (AlClPc) after loading it into chitosan (CHT)/chondroitin sulphate (CS) polyelectrolyte complexes (PECs) coated with polystyrene-*b*-poly (acrylic acid) (PS-*b*-PAA) nanoparticles (NPs). The NPs were cytocompatible upon healthy VERO cells and cytotoxic against colorectal cancerous cells.

In addition, Keyal et al. [57] investigated whether ZnPc-loaded chitosan/mPEG-PLA NPs topically applied onto tumor surfaces could increase penetration of PS through the keratinized surface of nodular tumors. In vivo studies performed with cutaneous squamous cell carcinoma tumor-bearing mice demonstrated that ZnPc-loaded nanoparticles possessed superior tumor accumulation and lack of systemic cytotoxicity compared to free ZnPc, indicating that the encapsulation of PS into nanocarriers and their subsequent topical application may be a promising platform for the treatment of skin cancers using PDT.

### 5.4. Polymeric Nanocarriers in PDT with Assistance of UCNPs

One of the drawbacks of PDT is that visible light has low penetration into deep-seated tumors. Currently the irradiating ability of many PSs is below 700 nm, which provides a light penetration of only a few millimeters from the tissue surface. The progress in the development of upconversion nanoparticles (UCNPs), which are capable of converting IR light into UV visible light, allow treatment of tumors that are not accessible to the visible light in PDT [137].

**Scheme 11:**
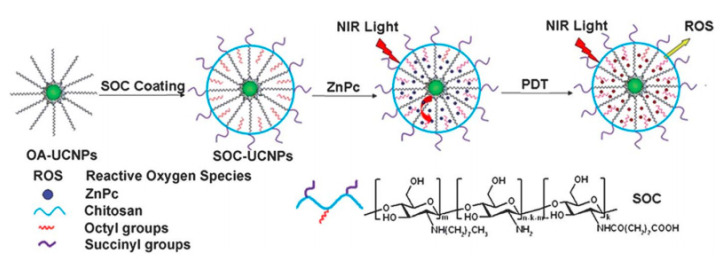
Schematic illustration for the synthesis of ZnPc-loaded SOC-UCNPs. Reprinted with permission from ref [81]. Copyright 2021 The Royal Society of Chemistry.

As an example of PDT-mediated cancer therapy based on the upconversion mechanism, the group of Gui et al. [81] coated UCNPs with amphiphilic chitosan (SOC) and loaded them with ZnPc (SOC-UCNP-ZnPc, Scheme 11). The average size of UCNPs was 35 nm and the thickness of the ZnPc-loaded layer of SOC was about 10 nm. In this way, ZnPc was close enough to the UCNPs, thereby facilitating resonance energy transfer from UCNPs to ZnPc. Surface modification with natural and biodegradable SOC enhanced the biocompatibility and decreased the toxicity of the UCNPs, and additionally served as a layer to carry PS into tumor cells. This material has great potential for PDT treatment in deep-seated tumors due to the deep tissue penetration of NIR light.

In 2013, the same group [82] prepared similar nanocarriers consisting of UCNPs; this time, in order to have better tumor targeting, they covered them with a folate-modified amphiphilic chitosan (FA-SOC) layer loaded with ZnPc (FA-SOC-UCNP-ZnPc). They proved that the ROS generation in cancer cells was higher upon excitation of UCNPs with the 980 nm light than that with 660 nm irradiation. For active targeting, they used folic acid (FA), which has a high affinity to folate receptors overexpressed in many cancer cells. Thus, the folate-modified surface enhanced the tumor-selectivity of the nanoconstructs to cancer cells that overexpressed folate receptors. They also observed low toxicity and fewer side effects at high doses (<150 mg/kg) in the mice verified by histological and biochemical analysis.

In the last example from this group [83] they modified the above system by preparing UCNPs coated with SOC with targeting ligand c(RGDyK) (c(RGDyK)-SOC-UCNP-ZnPc). Integrin a_v_β_3_ is overexpressed in a number of tumor types and is one of the key players in inhibiting tumor growth, tumor angiogenesis, and metastasis [138]. Moreover, it is overexpressed on tumor vascular endothelial cells but is not present in most healthy cells, making it a potential platform for targeted cancer therapy. Integrin α_v_β_3_ binds a wide range of extracellular matrix molecules with an Arg-Gly-Asp (RGD) triple-peptide motif, thus the group of Gu et al. [83] used c(RGDyK) ligand to facilitate active targeting of the NPs to tumor vasculature. 

Again, similar to the previous nanomaterials [81,82], after in vitro and in vivo studies the new one exhibited high photosensitizer loading capacity, selective targeting of the tumor vasculature, reduced toxicity after SOC coating, and conversion of NIR light into UV-visible light for deep-tissue PDT treatment. Additionally, they applied a two-step treatment strategy involving PDT and subsequent Doxil injection, resulting in a tumor inhibition rate of 79% compared with 56% after Doxil treatment alone in tumor-bearing mice. Also, the cardiotoxicity of Doxil was reduced compared to Doxil treatment alone.

All of these examples proved that PDT-mediated cancer therapy based on the upconversion nanoparticles could be an effective strategy to enhance the therapeutic efficacy of PSs during PDT.

### 5.5. Pc-Polymeric Nanocarriers in Combination of PDT with Chemotherapy

Combination therapies for cancer treatment could achieve either additive or synergistic effects arising from the action of two drugs with the final goal of maximization the therapeutic efficacy. The combination of PDT with chemotherapy could improve the cancer treatment by the simultaneous effect of the two drugs. There are many different ways in which PS could join forces with chemotherapy drugs against cancers. For example, it could be covalently conjugated to chemo drugs as the conjugation of sulfonated aluminum phthalocyanine and doxorubicin [139], or a combination of silicon phthalocyanine and paclitaxel [140]. Additionally, thanks to nanoscience, both anticancer agents could be entrapped into different types of nanocarriers. Here, the focus will be only on PS in combination with chemo drugs in polymer-based nanomaterials.

PEO_2000_–PCL_4300_ diblock copolymer and PEO_2000_–PCL_6800_–PEO_2000_ triblock copolymer exhibit amphiphilic properties and the tendency to form solid-like semicrystalline structures. Thus, they were used to prepare biodegradable core-shell NPs for the delivery of docetaxel and ZnPc into cancer cells [76]. PEO:PCL molecular weight ratio is a key determinant for stability in biological media. Hence, the NPs were chosen instead of micelles because micelles build of PEO-PLC copolymer could show disassembly in the bloodstream which could result in an activity profile of drug-PEO-PLC micelles similar to free drug [76]. The cytotoxicity of new NPs was evaluated in HeLa cells showing that the viability of cells treated with double-loaded NPs significantly decreased as compared to NPs loaded only with DTX. The same activity of ZnPc/DTX-loaded NPs was observed in an animal model of orthotopic amelanotic melanoma. The application of these novel PEO–PCL NPs in the combined chemo-photodynamic therapy of cancer could be very promising for synergistic therapy.

In addition, Dag et al. [77] prepared polymeric nanoparticles with ZnPc functionality, a pH-triggered cleavable core for Dox, and fructose-bearing glycopolymer on the surface of the NPs as a targeting ligand. The new nanocarriers presented superior uptake and higher cytotoxicity in vitro for human breast cancer cells. In vitro results proved that these dual-NPs had an enhanced chemo-PDT synergistic effect for breast cancer cell lines compared to chemotherapy alone or PDT alone.

**Scheme 12:**
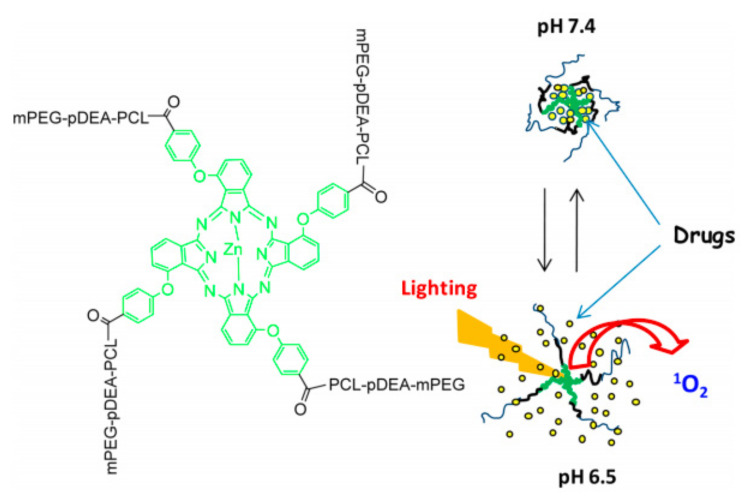
Schematic illustration of PDCZP and action of its pH-dependent drug release and combined therapy of chemotherapy and PDT. Reprinted with permission from ref [78]. Copyright 2017 American Chemical Society.

DOX was also used as the chemotherapeutic agent in four-armed star-shaped copolymer based on ZnPc as the PS (Scheme 12). The copolymer presented a tendency to self-assemble into DOX-loaded micelles [78], which had high tumor targeting and anticancer effects thanks to a combination of the two therapies. The pH-responsive photosensitizer-core four-armed star-shaped copolymer, [methoxy-poly(ethylene glycol)-poly(2-(N,N-diethylamino)ethyl methacrylate)-poly(ε-caprolactone)]4-zinc β-tetra-(4-carboxyl benzyloxyl)phthalocyanine (PDCZP) was successfully designed and prepared. Interestingly, in aqueous media the PDCZP assemble into spherical nanocarriers with pH dependent size (51 nm at pH 7.4, 105 nm at pH 6.5, and 342 nm at pH 5.0). Moreover, after doxorubicin loading, the nanocarriers showed better in vitro and in vivo anticancer effects under light irradiation on human breast cancer MCF-7 cells, human colon cancer SW480 cells, human hepatocellular cancer HepG2 cells, and H22 tumor-bearing mice than each therapy alone.

In the last example, Yue and co-workers [84] developed the strategy of targeting mitochondria, which finally maximizes the photodynamic therapeutic efficiency for cancer by using ZnPc/CPT-TPPNPs in PDT of NCI-H460 human lung cancer cells. First, polyethylene glycol was modified with thioketal linker-functionalized camptothecin (TL-CPT) and triphenylphosphonium, which resulted in the block copolymer (TL-CPT-PEG1K-TPP). The ZnPc/CPT-TPPNPs were prepared by blending the block copolymer TL-CPT-PEG1K-TPP with 1, 2-distearoyl-sn-glycero-3-phosphoethanolamine-N-[methoxy(polyethylene glycol)] (DSPE-PEG). Furthermore, the NPs consisted of mitochondria-targeting small molecule PPh_3_Br-(CH_2_)_4_-COOH (TPP). Not only were the final NPs able to target mitochondria but camptothecin (the chemo-drug, CPT) release was also exclusively ROS-responsive thanks to the presence of ROS-responsive thioketal linker. Additionally, NPs had a positive charge which permitted their transport through the lipid bilayers and to accumulate selectively in mitochondria.

### 5.6. Bioresponsive Pc-Polymeric Nanocarriers in PDT

Recently, many studies have focused on the design of new bioresponsive nanocarriers which, under the action of particular endogenous or exogenous stimuli, release the PS directly into the tumor tissues in a controlled spatiotemporal manner. After the incorporating of various stimuli-responsive groups into the copolymer structure, the resulting polymeric nanocarriers can respond to an endogenous stimulus (e.g., ROS, pH, temperature) or exogenous stimulus (e.g., light, ultrasound, magnetic field,) by swelling/shrinking, disassembly/reassembly, surface, charge conversion, etc., resulting in the on-demand release of encapsulated PS in a pathologically changed place and significantly improving selectivity and efficacy of PDT [141]. As known, the tumor microenvironment significantly differs from the healthy tissues. The cancerous tissues are characterized by various characteristic pathophysiological markers, e.g., acidic intratumoral and endosomal pH, elevated intracellular ROS or glutathione level, overexpression of specific enzymes (e.g., Cathepsins, MMP-9), or hypoxia conditions [142]. Thus, these factors could act as an endogenous stimulus for the release of encapsulated PS in the tumor tissues, increasing the PDT efficacy without damaging normal tissues. The acidic intracellular and extracellular pH of tumor tissues constitutes a proper endogenous stimulus in designing tumor-targeting nanocarriers. The acidic intra- and extracellular pH of tumor tissues resulting from the unique metabolism of sugars by cancer cells (Warburg effect) and/or an increased level of glutathione are promising endogenous stimuli in the design of tumor-targeting nanocarriers. 

**Scheme 13:**
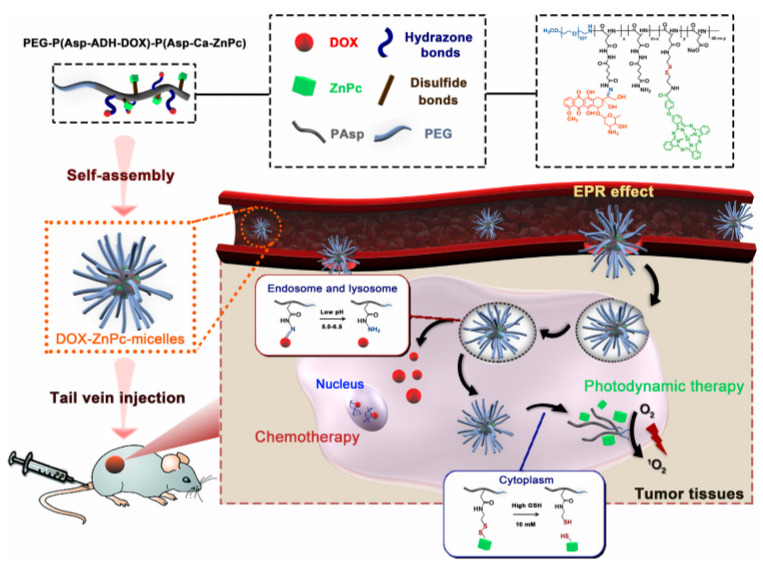
Schematic diagram showing the structure of the pH- and redox-responsive prodrug DOX-ZnPc-micelles and their mechanistic action for chemotherapy and PDT. Reprinted with permission from ref [85]. Copyright 2018 Elsevier.

Taking advantage of these factors, Gao et al. [85] designed and synthesized micelles consisting of PEGylated poly(β-benzyl-L-aspartate), in which doxorubicin and ZnPc were conjugated to copolymer via a pH-responsive hydrazine bond and glutathione-sensitive disulfide bond, respectively (Scheme 13). The drug release studies showed pH-responsive DOX release and glutathione-triggered ZnPc release, while in vitro cellular studies revealed that micelles exhibited a synergistic effect in inhibiting HepG2 cell growth. In vivo studies on tumor-bearing nude mice showed that the nanosystem with a DOX/ZnPc molar ratio of 3.8 was the most effective, causing inhibition of tumor growth due to DOX-induced chemotoxicity and the ROS generated upon irradiation of the ZnPc.

Subsequently, the same team [143] successfully used this system to treat DOX-resistant human hepatocellular carcinoma cells. The enhanced cellular accumulation of doxorubicin in DOX-resistant-HepG2 cells by DOX/ZnPc-loaded micelles induced a higher cytotoxicity effect compared with free doxorubicin. Importantly, a combination of chemotherapy and phototherapy showed enhanced cytotoxicity synergistically. 

Very recently, the same group reported an elegant way for the coadministration of chemo and PDT agents. The DOX and ZnPc were linked via a pH-sensitive hydrazone bond. The resulting prodrug and tirapazamine (a hypoxia-activated anticancer drug) were subsequently loaded into poly(ethylene glycol)-*b*-poly(D, L-lactide) micelles. The CLSM studies showed an internalization efficiency, followed by cleavage of the hydrazone linkage in ZnPc–Dox prodrug in the tumor acidic intracellular microenvironment. In addition, cellular studies revealed that a combination of chemo- and photodynamic therapy displays superior therapeutic outcomes compared to bare chemotherapy or PDT [144].

Breitenbach et al. [86] designed reduction-/pH-sensitive nanocarriers based on amphiphilic copolymer consisting of acetalated dextran and dextran blocks linked via a disulfide bond (dextran-*block*-acetalated dextran, Dex-*b*-AcDex). Amphiphilic copolymer formed micelles with an average size of ~120 nm and were loaded with ZnPc. The acetal groups hydrolyzed in an acidic intra- and extracellular tumor microenvironment, resulting in increased PS release. Subsequently, the internalized micelles were disintegrated because of disulfide linkage cleaving due to a high level of glutathione, resulting in the rapid release of loaded PS. In vitro drug release studies revealed that at pH 5 or in the presence of reduction agent (dithiothreitol), ZnPc was significantly accelerated. Furthermore, these ZnPc-loaded dual-sensitive nanocarriers accumulate in cancer cells, leading to efficient inhibition of proliferation upon irradiation with NIR light. 

Tumor tissues are also characterized by higher temperatures than healthy tissues due to the increased metabolic rate. Therefore, a few thermo-responsive nanoparticles have been proposed as PDT agent nanocarriers. To illustrate this, Rijcken et al. [145] demonstrated thermo-sensitive nanoparticles consisting of poly(ethylene glycol)-*b*-poly(N-(2-hydroxypropyl)methacrylamide-dilactate) via controlled radical polymerization. Subsequently, such prepared nanoparticles were loaded with axially solketal-substituted silicon phthalocyanine and exhibited the temperature-dependent drug release and enhanced cellular uptake, which was confirmed by confocal laser scanning microscopy studies.

Furthermore, Li et al. [87] described silicon(IV) phthalocyanine dichloride (SiPc) loaded PEGylated-methacrylates-based temperature-responsive nanocarriers for tumor-targeted PDT. Respectively, poly(OEGMA-*co*-DEGMA-*co*-HEMA) was synthesized by RAFT polymerization of the oligo(ethylene glycol) methyl ether methacrylate (OEGMA), di(ethylene glycol) methyl ether methacrylate (DEGMA), and 2-hydroxyethyl methacrylate (HEMA) monomers. The lower critical solution temperature of the copolymer was controlled by changing the ratio of the applied monomers. This nanosystem displayed a similar singlet oxygen quantum yield in DMF (ΦΔ = 0.55) compared to free ZnPc (ΦΔ = 0.56).

Additionally, Liu et al. [88] reported microgel particles consisting of poly(N-isopropylacrylamide)/lipid (pNIPAM/lipid) composite for thermo-responsive release of PDT agents. Microgel particles have emerged as an interesting system for drug delivery because they combine the unique properties of a gel with those of micro-/nanoparticles. Thus, in this work, the particles were loaded with SiPc. The addition of lipid (dipalmitoylphosphatidylcholine) molecules increased the PS loading properties compared to free pNIPAM-based nanocarriers. In vitro cellular studies revealed that SiPc-loaded pNIPAM/lipid composite was internalized into HeLa cells, which upon light irradiation kill cancer cells due to the PDT effect of the PS. 

In this context, Li et al. [89] demonstrated a thermo-sensitive copolymer poly(ethylene glycol)-*b*-poly(N-isopropylacrylamide) (PEG-*b*-PNIPAAM) functionalized with zinc(II) tetraaminophthalocyanine (ZnTAPc) as an end group. The micelles with an average size of ~45 nm formed from this amphiphilic copolymer possessed adjustable lower critical solution temperature (LCST) between body temperature (37 °C) and tumoral hyperthermia (42 °C). The LCST of MPEG24-*b*-PNIPAAM96-ZnTAPc was 41.6 °C, which is suitable for targeted aggregation at the tumor site with mild local hyperthermia. This system generates singlet oxygen species with good quantum yields (ΦΔ = 0.56), while MTT assays showed a lack of dark cytotoxicity and good phototoxicity effect on cancer cells (HeLa).

Feuser et al. [90] developed poly(methyl methacrylate) (PMMA) based nanocarriers combining the delivery of ZnPc in PDT and photothermal therapy (iron oxide magnetic NPs). The ZnPc were used in PDT under laser irradiation and magnetic nanoparticles for the hyperthermia effect under an alternating magnetic field. The utility of the dual-loaded nanocarriers was tested against human glioblastoma cells, wherein it was found that the application of the light dose and magnetic field together exhibited a synergistic effect for inhibiting the proliferation of cancer cells as against either of the two treatments applied separately. The tumors are heterogeneous, showing different levels of pathophysiological markers in their cells. Therefore, the utilization of exogenous-stimuli-responsive nanocarriers (e.g., responsive to a magnetic field or light) are a highly attractive strategy for overcoming tumor heterogeneities, thanks to their non-invasiveness, accuracy, and ability to achieve on-demand release of PS in a spatiotemporal manner [146].

**Scheme 14:**
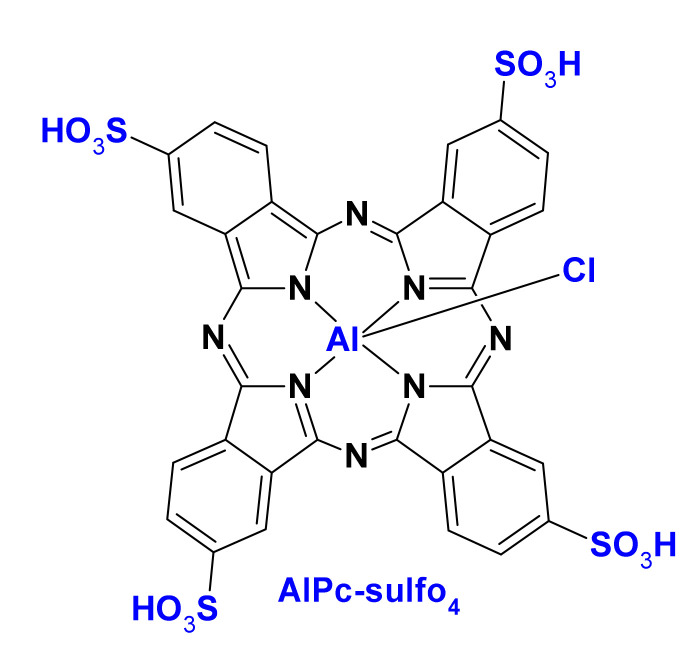
The structures of AlPc-sulfo_4_.

Similarly, S. Duchi et al. [91] improved PDT of prostate cancer by using PMMA loaded with the PS tetrasulfonated aluminum phthalocyanine (AlPc-sulfo_4_, Scheme 14) and fluorescein derivative (FITC). This new nanosystem was successfully internalized by tumor cells and efficiently triggered cancer cell death after irradiation with 680 nm light. After in vivo experiments, NPs significantly reduced the tumor growth with higher efficiency than the bare Pc. Thus, these NPs could be utilized as a delivery system for solid cancer PDT.

Recently, a series of NIR-activatable nanocarriers based poly-L-glutamic acid with Pc conjugates in a side chain has been reported for cancer PDT [147,148]. These nanosystems possessed an excellent light-dark toxicity ratio compared to free ZnPc. In vitro studies revealed high NIR light absorptivity and enhanced cellular uptake, leading to a superior phototoxicity effect compared to free PS. Moreover, in vivo studies showed that a fourfold lower dose of ZnPc-loaded nanocarriers still caused greater tumor volume reduction when compared to free ZnPc.

**Scheme 15:**
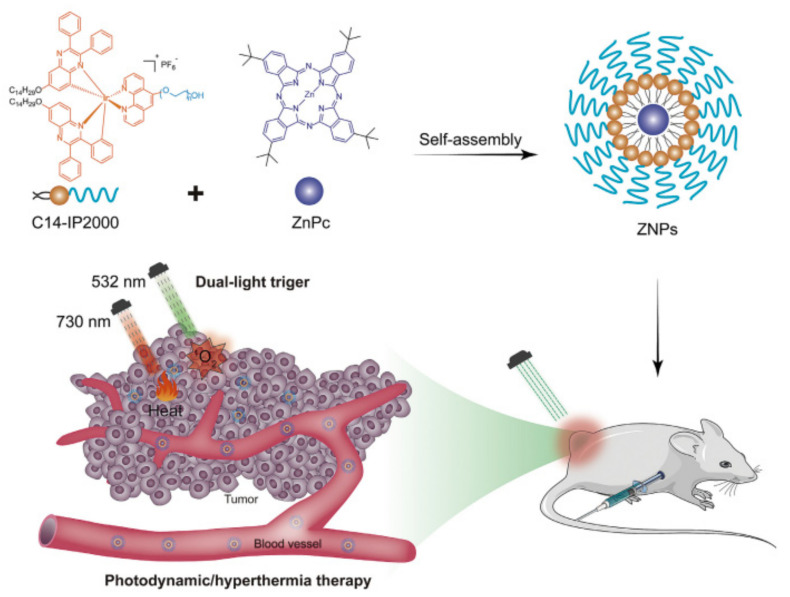
Schematic illustration of ZnPc encapsulated amphiphilic Ir(III) complex nano-micelle for selective tumor imaging and dual-light triggered combination therapy. Reprinted with permission from ref [149]. Copyright 2021 Elsevier.

Very recently, Deng et al. [149] designed a smart dual-light triggered nanocarrier for tumor-targeted P/hyperthermia therapy (Scheme 15). The amphiphilic iridium-based photosensitizer (C14-IP2000) was mixed with a photothermal drug (ZnPc) to form micelles with an average size of ~55 nm. Upon dual-light irradiation, i.e., 532 nm and 730 nm, the nanosystem generated the singlet oxygen species and induced high heat, showing successfully inhibited tumor proliferation and decreased tumor volume in mice bearing 4T1.2 tumors. Meanwhile, ex vivo studies showed negligible adverse effects in major organs and good biocompatibility. Thus, a combination of PDT with PTT could be a promising approach in clinical applications for combination tumor therapy.

## 6. Conclusions

In the past decade, considerable progress has been made in the synthesis and characterization of Pc-polymeric NP delivery systems and their applications in cancer PDT. Introduction of Pcs into polymeric NPs can improve their photophysical properties and selectivity for targeted tissues, and not only eliminate aggregation of the PSs due to their low water solubility but also enhance treatment by increasing blood circulation and selective accumulation in tumor tissues due to the EPR effect. This article reviewed the recent progress in Pc-polymeric NPs for enhancement of cancer PDT. Although there are many achievements in this field, further research is needed to prepare biocompatible and selective targeting PS for cancer PDT. For instance, the PDT efficacy of nanomedicines could be further improved by (1) developing new PS which provide higher ^1^O_2_ generation properties compared to those which already exist; (2) preparing nanomaterials with better selectivity for targeted cancer tissues; (3) combining other cancer therapies with PDT (e.g., chemotherapy, surgery); (4) delivering additional oxygen to solid tumors or generating it in vivo, in order to provide a continuous local oxygen supply which enhances PDT efficiency; and (5) investigating ways to improve equipment for the delivery of activating light, which can penetrate tissue to treat deep or large tumors. With all of these challenges to address, improving the use of nanocarriers in PDT could become a powerful strategy for use in urgent cancer therapy.

## Figures and Tables

**Table 1:** The examples of Pcs used in PDT (1) currently under investigation in clinical trials, (2) already approved for clinical use, and (3) appeared in this review in polymeric nanoparticle delivery systems. Maximum absorption (λ_max_); ^1^O_2_ quantum yield (Φ_Δ_).

Name	λ_max_ (nm)	Φ_Δ_	Cancer Type/Cell Line/Animal Model
Photosens (sulfonated AlPcs) [14,26]	676 (DMF)	0.38 (DMF)	skin, stomach, lip, breast, and oral cancer (2)
Pc 4 (silicon phthalocyanine 4) [14,26]	675 (CH_3_CN)	0.43 [27] (CH_3_CN)	actinic keratosis, Bowen’s disease, skin cancer, mycosis fungoides (1)
Photocyanine [22,28]	675 (DMSO)	0.53 (DMSO)	HepG2 (1)
ZnPc [29,30,31]	672 (DMSO)	0.67 (DMSO)	cutaneous and subcutaneous lesions from diverse solid tumor origins (1,3)
AlPc [32,33]	680 (DMSO)	0.29 (DMSO)	J774A.1, Vero cells [34] (3)
RuPc-(4–12 PEG) [35]	638–642 (H_2_O/DMSO 99: 1)	0.76 (DMSO)	HT-1376 (3)
PEG-ZnPc [36]	679–686 (DMF)	-	HEp2 (3)
Tetraethyleneglycol-substituted ZnPc [29]	676–702 (DMSO)	0.34–0.72 (DMSO)	HT-29 (3)
Mono-PEGylated ZnPc [37]	672 (DMF0	0.53–0.56 (DMF)	HepG2 (3)
ZnPcBCH_3_ [38]	681 (DMF)	0.51 (DMSO)	A549 (3)
Poly(aryl benzyl ether)dendrimer ZnPc [39]	620–630 (PBS)	0.43–0.56 (DMSO)	HeLa (3)
Amphiphilic SiPc deriv [40].	686 (CHCl_3_)	0.27 (DMSO)	RAW 264.7 (3)
Benzyl ester dendrimer SiPc [41]	678 (DMF)	0.31 (DMF)	-
NzPc [42]	682 (EtOH)	0.63 (EtOH)	-

**Table 2:** The examples of Pc-polymer nanomedicines appearing in the literature since 2010 (M—micelle, NP—nanoparticle).

Material Description	Nanocarrier Type	Pc-Type	NP Size [nm]	Cell Line/Animal Model	Active Targeting
PLGA [49]	NP	ZnPc	285 ± 5.1	P388-D1	-
PLGA-PEG [50]	NP	InPc	61–243	MCF-7	-
PLGA-PEG [51]	NP	GaPc	>200	Hepa-1C1C7, blood red cell	-
PEG-*b*-PLGA [38]	NP	ZnPcBCH_3_	90.02 ± 0.07	A549	-
PEG-PLA-BGE [34]	M	AlPc	60–130	J774A.1, Vero cells	-
PLGA [52]	M	ZnPc-sulfo_4_	384.7 ± 138.6	B16-F10	-
PLGA-HA [53]	NP	ZnPc-	259	HT29, A549, LO2/HT29 tumor-bearing nude mice	HA
PEG-*b*-PLLA [54]	M	ZnPc	32–35	Me45, HaCaT, P388/D1, HUV-EC-C	-
FA-PEG-*b*-PLLA [55]	M	ZnPc	<150	SKOV3, Me45	FA
ZnPc-PEGylated Pluronic P123/PLLA [56]	M	ZnPc	15–89	MeWo	-
chitosan/mPEG-PLA [57]	NP	ZnPc	189.7–3.5	SCC, A431/SKH-1 hairless mice	-
PEG-*b*-PCL [58]	NP	ZnPc	60	SC	HPβCD
PEG-*b*-PCL [40]	M	SiPc deriv.	-	RAW 264.7	-
PEG-*b*-PCL [59]	M	SiPc/ZnPc deriv.	111/77	MCF-7	-
PEG-*b*-PCL [60]	M	AlPc	66.5–99.1	female Balb/c mice	
Pluronic F127 [61]	M	AlPc	6	A549	-
Pluronic F127 [62]	M	InPc/ZnPc deriv.	27.1–37.8	MCF-7	-
Tetronic 1107 [63]	M	ZnPc deriv.	10–100	CT26	-
Pluronic F127 [64]	M	4OCSPC	193.2	HeLa/mice bearing 4T1 tumor	-
Pluronic F127, pNIPAM [65]	M	4OCSPC	193.2	HeLa	-
PMMA [66]	NP	ZnPc	97 ± 2.5	L929, HPBL, K562, Jurkat	-
PSt-*b*-PPEGA [67]	M	ZnPc	190–210	HeLa	-
P(R)-*b*-PPEGA [68]	M	ZnPc	167–230	RGK-1	-
PS-*b*-PAA [69]	M	AlPc	139.9 ± 0.8	Caco-2	-
PEG-PMAN [70]	M	ZnPc	30	MNNG/Hos, U2OS, Saos-2, MG-63/subcutaneous mouse	-
PEG-PCL [71,72,73]	M	Pc 4	80–100	A431, MCF-7c3	EGFR
PEG-*b*-PCL [25]	M	BtPc	95–110	HeLa cells	-
PEG-*b*-PCL [74]	M	SiPc deriv.	45–70	-	-
PLL-*b*-PEG-*b*-PLL, PEG-*b*-PLL [75]	M	S-AlPc	10–70	HUVECs	-
PEG_5000_-*b*-PLA_3000_ [41]	M	D-SiPc	100	U251	-
PLL-*b*-PEG-*b*-PLL [39]	M	ZnPc-dendrimers	80–150	HeLa	-
PLGA [42]	NP	NzPc	435	WS-21	-
PEO_2000_–*b*-PCL_4300_ PEO_2000_–PCL_6800_–*b*-PEO_2000_ [76]	NP	ZnPc/DTX	60–100	HeLa	-
P(MMA-*b*-MAEBA-*b*-FrucMA)-ZnPc/Dox [77]	NP	ZnPc/DTX	30	3T3, MCF7, MDA-MB-231	GLUT5
mPEG-pDEA-PCL)_4_-ZnPc_4_ (PDCZP) [78]	M	PDCZP	51–342	MCF-7, SW480, HepG2/H22 tumor-bearing mice	pH
SOC, PCL [79]	NP	ZnPc	100	-	
phosphonium chitosan [80]	M	ZnPc	103 ± 5	Panc-1	
SOC with UPNPs [81]	NP	ZnPc	45	HELF, MCF-7/S180 tumor-bearing mice	
folate-modified SOC UPNPs [82]	NP	ZnPc	50	HELF, MDA-MB-231/S180 tumor-bearing mice, Bel-7402 tumor bearing mice	FA
c(RGDyK) modified SOC with UPNPs [83]	NP	ZnPc	52	PC-3, WPMY-1/PC-3 tumor-bearing mice	α_v_β_3_
TL-CPT-PEG1K-TPP [84]	N	ZnPc/CPT	77.1–149.	NCI-H460/Female BALB/c athymic nude mice	n
PEG-*b*-PBLA [85]	M	ZnPc/Dox	160–180	HepG2/HepG2 tumor-bearing nude mice	GSH
Dex-*b*-AcDex [86]	M	ZnPc	120	HeLa	pH
poly(OEGMA-*co*-DEGMA-*co*-HEMA) [87]	M	SiPc	70	-	temp.
pNIPAM/lipid [88]	microgelparticles	SiPc	1000	HeLa	temp.
PEG-*b*-PNIPAAM [89]	M	ZnTAPc	45	HeLa	temp.
PMMA, MNP [90]	NP	ZnPc	104 ± 2.5	U87MG	magnetic field
PMMA [91]	NP	AlPc-sulfo4	80	MSC, PC3, SCID mice withPC3	-

## Abbreviations

λ_max_	maximum absorption
Φ_Δ_	singlet oxygen quantum yield
AlPc	aluminum-phthalocyanine chloride
AlPc-sulfo_4_	tetrasulfonated aluminum phthalocyanine
CPT	camptothecin
DEGMA	di(ethylene glycol) methyl ether methacrylate
Dex-*b*-AcDex	dextran-block-acetalated dextran
DOX	doxorubicin
DTT	dithiothreitol
FDA	Food and Drug Administration
GaPc	gallium (III) phthalocyanine chloride
GSH	glutathione
HEMA	2-hydroxyethyl methacrylate
InPc	indium(III) phthalocyanine chloride
MNP	magnetic nanoparticle
NP	nanoparticle
PEG-*b*-PLLA	poly(L-lactide)-*b*-poly(ethylene oxide) block copolymer
PEG-*b*-PNIPAAM	poly(ethylene glycol)-*b*-poly(N-isopropylacrylamide)
OEGMA	oligo(ethylene glycol) methyl ether methacrylate
PAH	polyalkylamine hydrochloride
PBLA	poly(β-benzyl-L-aspartate)
Pc	phthalocyanine
PCI	photochemical internalization
PDT	photodynamic therapy
PEG	polyethylene glycol
PEG-*b*-PCL	poly(ethylene glycol)-*b*-poly(ε-caprolactone) diblock copolymer
PEG-PMAN	poly(ethylene glycol)-poly[2-(methylacryloyl)ethylnicotinate]
PEG-*b*-PLGA	poly(ethylene glycol)-*b*-poly(lactide-*co*-glycolide)
PMMA	poly(methyl methacrylate)
pNIPAM	poly(N-isopropylacrylamide)
P(R)-*b*-PPEGA	poly(N-substituted acrylamide)-*b*-poly(polyethylene glycol monomethyl ether acrylate)
PS	photosensitizer
PS-*b*-PAA	poly(styrene)-*b*-poly(acrylic acid)
PSt-*b*-PPEGA	polystyrene-*b*-poly(polyethylene glycol monomethyl ether acrylate)
PSS	poly(4-styrene sulfonate)
PTT	photothermal therapy
RuPc(4–12 PEG)	(ruthenium(II) phthalocyanines functionalized with 4–12 PEG chains
SiPc	silicon (IV) phthalocyanine dichloride
ZnPc	zinc (II) phthalocyanine
ZnPcBCH3	2(3), 9(10), 16(17), 23(24)-tetrakis-(4′-methyl-benzyloxy) phthalocyanine zinc(II)
ZnPcF_16_	zinc 1,2,3,4,8,9,10,11,15,16,17,18,22,23,24,25-hexadecafluoro29H,31H-phthalocyanine
ZnPc-sulfo_4_	Zinc(II) phthalocyanine tetrasulfonic acid
ZnTAPc	zinc(II) tetra-aminophthalocyanine
